# Impact of Social Media on Skin Cancer Prevention

**DOI:** 10.3390/ijerph18095002

**Published:** 2021-05-09

**Authors:** Henriette De La Garza, Mayra B. C. Maymone, Neelam A. Vashi

**Affiliations:** 1 Department of Dermatology, Boston University School of Medicine, Boston, MA 02118, USA; hadelaga@bu.edu; 2 Department of Surgery, University of Colorado School of Medicine, Aurora, CO 80045, USA; mayrabcm@gmail.com

**Keywords:** dermatology, social media, skin cancer, sun damage, tanning, Instagram, Twitter, Facebook, YouTube, TikTok, influencers

## Abstract

Despite the increasing prevalence of social media usage in health care contexts, its impact on skin cancer prevention and awareness has not been largely investigated. We conducted a review of literature on this topic with the objective of summarizing and analyzing the role of social media in skin cancer and sun damage awareness and to identify the uses, benefits, and limitations of different social media platforms on skin cancer prevention. In today’s technological society, it is critical to understand and study the best form of communication. Specific platforms like Instagram, Twitter, Facebook, YouTube, and TikTok vary in originators of material, target demographics, messaging strategies, and reliability of information with regards to skin cancer, sun, and indoor tanning damage. Our results demonstrate that social media interventions have shown promise in skin cancer prevention and continue to escalate by the day. Dermatologists should keep pace with the latest dermatological content on social media and examine its evolution to target the right audience with the proper messages. Further research is needed to evaluate the effectiveness and true impact of social media on meaningful and lasting behavior change for skin cancer prevention.

## 1. Introduction

Social media has impacted people’s daily lives in ways no one ever thought possible. In 2021, there were 4.20 billion social media users around the world, equating to more than 53% of the total global population [1]. There is an ongoing increase in the use of social media, including in health care contexts. Patients and health professionals are using these platforms to communicate and seek information about a wide variety of health issues. The ubiquitous use of social media has led physicians to envision ways in which social media can promote better health outcomes. Social media platforms constitute a powerful means of communication that can be used to educate patients and elevate public awareness of many diseases and health issues.

Social media is changing the nature and speed of health care interaction between individuals and health care professionals. In the United States, 61% of adults search online and 39% use social media platforms for health information [2]. There have been many applications of social media within health contexts, ranging from the World Health Organization (WHO) using Twitter during the influenza A (H1N1) pandemic, with more than 11,700 followers, to health professionals promoting information about their clinical practice [2].

The focus of these initiatives is represented by awareness-building campaigns and marketing that take advantage of the immediate communication and broad reach of social media. These campaigns often convert knowledge and information into discussions and conversations, which help to reach more people and promote further education and healthy behaviors. Social media platforms are a cost-effective and efficient way to create and publish information [3].

Social media represents an understudied opportunity for skin cancer prevention. Currently, there is a lack of information about the uses, benefits, and limitations of social media for skin cancer prevention, although several studies have investigated its utility and significant potential has been demonstrated [4]. Given the profound influence and increasing use of social media and the emerging evidence of its effects on human behavior, this can be a powerful tool to promote skin cancer awareness and sun protection.

The objective of this paper was to review the current literature to summarize and analyze the role of social media in skin cancer and sun damage awareness, and to identify the uses, benefits, and limitations of different social media platforms on skin cancer prevention. In order to establish whether social media improves health communication practices related to skin cancer.

## 2. Methods

An electronic search was undertaken on PubMed database in March 2021. The search terms “social media” and “skin cancer” were used. The time span was set as “all years”, producing 110 total results. For the purpose of this literature review, only studies investigating trends related to skin cancer awareness and the way people seek out skin cancer information in social media were included. The relevance of retrieved articles was evaluated using the following inclusion criteria:

- Research focused on the observations of people’s behaviors reflected on one or more specific social media platforms (Instagram, Twitter, Facebook, YouTube, and TikTok) and/or the investigation of people’s perceptions on seeking skin cancer information from social media.
- Focus on dermatologist perspectives and/or approach of skin cancer prevention through social media platforms.
- Focus on communication interactions between the general public and/or patients and/or health professionals about skin cancer using social media.
- Findings that identified the advantages and/or disadvantages of looking for skin cancer information in social media from both consumer and dermatologist perspectives.

The exclusion criteria were:

- Studies not in English.
- Studies that evaluated certain online health-related websites or other platforms that were not Instagram, Twitter, Facebook, YouTube, or TikTok.

The results were screened by title and abstract to establish relevancy. Each paper was checked by the authors, identifying 20 papers as relevant and meeting the inclusion criteria. The full-text studies that met the criteria listed above were further reviewed. The authors extracted data mainly from the results, discussion, and conclusion parts of each included article.

## 3. Results 

Among the 110 screened articles, 20 final articles met the inclusion criteria. These articles were published between 2014 and 2021. Table 1 summarizes the characteristics of the examined papers in terms of type of article, targeted issue, and social media discussed. The majority of the articles (25%) were letters to the editor, followed by original articles (20%). Fifty percent of the research papers concentrated on the exploration of one or more specific issues including primarily skin cancer and indoor tanning, followed by tanning attitudes, melanoma, sun damage, sun-related behaviors, tanning bed burns, tanning health risks, sun protection, and sunburn. Five types of social media platforms were included: Instagram, Twitter, Facebook, YouTube, and TikTok. Among the included studies, 25% of the papers targeted one or more specific social media platforms. Twitter and Facebook were investigated the most, followed by Instagram, YouTube, and TikTok. Table 2 highlights important statistics regarding article type, topics covered, topics covered by each social media platform, and most used social media platforms.

## 4. Discussion

### 4.1. Social Media in Dermatology

Social media has become a popular cost-effective way for public health practice to inform the public of health issues, enhance communication during health emergencies or outbreaks, and respond to public reporting of a particular health issue [3]. Social media represent a variety of platforms and ways to communicate information. A few years ago, this encompassed a simple post or image, which grew into the possibility of likes, reactions, and comments which increase the engagement of those publications. Today, the possibilities of publishing are infinite, with stories, reels, polls, question boxes, and live videos that unlike traditional methods such as broadcast news or posters, have the capacity to interact with the audience in an immediate way. The emerging presence of social media in dermatology provides opportunities for dermatologists to participate in dissemination of reliable health information, social networking, and inspiration. Dermatologists are increasingly using social media to share journal articles, promote professional opinions, and circulate information about their work, which also provides an opportunity to increase patient volume through both advertisements and word-of-mouth marketing.

Gantenbein et al. found that 82.4% reported searching the internet for medical information, and 65.4% reported to be users of social media [25]. Professional dermatology organizations and board-certified dermatologists have increased their public and professional engagement through social media. The use of social media has also gained recognition among the scientific community because its use is associated with higher article citation and increased accessibility to the general public [26]. In 2018, Patel et al. revealed that the two most popular dermatology journals on social media were the *Journal of the American Academy of Dermatology* (JAAD) and *JAMA Dermatology*. Currently, JAAD has remained the most popular on Facebook, with an increase in likes from 44,282 in 2017 to 75,707 in 2021. *JAMA Dermatology* is still the most popular on Twitter, with an increase in followers from 13,072 in 2017 to 19,300 in 2021 [27]. These compelling numbers are proof of the growing trend and usage of social media in the scientific community over the last few years. These platforms have become powerful instruments for health education that people from diverse age groups, locations, and economic statuses can use daily to learn and share knowledge [26]. Given this rise of dermatology content on social media, it is relevant to consider how social media is being used in regards to dermatological concerns and practices and how to best tackle them. 

### 4.2. Why Skin Cancer?

A person’s skin reflects the general health condition and its aging process. Exposure to excessive or harmful ultraviolet (UV) rays results in premature aging, initiation of reactive oxygen species generation, and skin cancer [28]. Skin health is perceived as an indicator of one’s beauty, and this has resulted in rising awareness of sun damage and interest in skin care as well as an increasing demand for sunscreen.

The prevention of skin cancer is of particular significance, as its incidence has risen dramatically in recent decades. Skin cancer, including squamous, basal, and melanoma, represent the most commonly diagnosed forms of cancer worldwide [5]. Melanoma is the third most common type of cancer among adolescents and young adults aged 15–39 years [29]. Non-melanoma skin cancers represent the majority of cutaneous malignancies and affect one in five Americans during their lifetime [30]. While the prognosis for most patients is excellent, significant morbidity and preoccupation may occur due to the predilection for these cancers to arise in cosmetically sensitive areas such as the face and neck. 

About 90% of non-melanoma skin cancers and 85% of melanoma cases are associated with exposure to UV radiation from the sun. Exposure to UV radiation from outdoor light (sun) and indoor light (tanning beds, lamps or booths) is the main cause of skin cancer, but is also the most preventable one. Surprisingly, many people are not aware of this. A survey from the Centers for Disease Control and Prevention (CDC) found that 50.1% of all adults and 65.6% of white adults ages 18–29 reported suffering sunburn in the past 12 months, indicating that sun protection measures are not followed correctly [31]. Similarly, Wehner et al. stated that the popularity of indoor tanning is alarming, particularly among young people. Their research revealed that 35% of adults had been exposed to indoor tanning, with 14% reporting tanning bed use in the past year. Even more of a concern was that 43% of university students and 18% of adolescents reported using tanning beds in the past year. The researchers estimated that overall rates of tanning bed use may lead to an additional 450,000 non-melanoma and 10,000 melanoma skin cancer cases every year [32]. According to a survey by ABC news, one-third of Americans don’t use sunscreen [33]. There is a tremendous gap in knowledge about proper sunscreen use. Questions remain about who should use it, when they should use it, and how often they should reapply it. 

Primary prevention efforts have focused on increasing awareness about the dangers of excessive UV exposure, but clearly there is an urgent need to emphasize and promote this message. Secondary prevention focuses on early detection and control of cancer, thereby leading to timely initiation of therapy and improved outcomes. Dermatologic consults and screening exams are the cornerstones of secondary prevention strategies. By identifying individuals with risk factors, screening allow for the detection of disease in asymptomatic individuals, which is why it is of utmost importance to encourage patients to seek dermatologic care [30]. 

Skin cancer is not only a major public health problem, but also a huge economic burden. The number of adults treated for skin cancer increased from 3.4 million between 2002–2006 to nearly 5 million between 2007–2011. Average annual total treatment costs for skin cancer also increased substantially between these periods to $8.1 billion annually [6]. The incidence of skin cancer has risen significantly due to increasing use of recreational tanning and the perceived social desirability of tanned skin. In the era of social media, people seek or gain inspiration through these platforms. The images on social media carry a very powerful message. Millions of people are influenced by what they see, be it a sculpted body, an expensive purse, or a perfectly tanned face. People want what they see, and this is why social media has also become a popular marketing tool. “Influencers” are individuals that have many followers on social media and receive payment or company products to use and advertise products, procedures, and places through their platforms. Influencers have the power to affect the attitudes and purchasing decisions of others because of their authority, knowledge, position, or relationship with their audience. However, it is important to note that many influencers do not have medical or educational backgrounds; hence, the information that they share may not always be accurate and/or can be misleading. Abbot et al. found that regular use of Instagram was significantly associated with increased odds of intentional tanning and feeling more attractive when tanned [7]. Similarly, Falzone et al. reported that higher rates of indoor tanning are associated with regular Instagram and Twitter use [8]. Celebrities also play a huge role, not only in the marketing business but also in influencing the behaviors of their followers. Celebrity health disclosures can lead to significant media coverage and public engagement. For instance, Angelina Jolie’s 2013 disclosure that she had undergone a prophylactic mastectomy resulted in large increases in online breast cancer search queries, followed by a near doubling of demand for BRCA1/2 testing [9]. This suggests that social media could be a key venue for reaching a large number of people with skin cancer prevention interventions. Nowadays, there is a gap in cancer prevention messages. While social media can perpetuate misinformation, it can also be used to combat misinformative messages. Therefore, it is of utmost importance to provide correct information about the effects of UV light on the skin, promote sun-safe behaviors, indoor and outdoor tanning cessation, and early detection of skin cancer.

### 4.3. Trends within Specific Social Media Platforms

#### 4.3.1. Instagram

With rising rates of social media use in dermatology, patients are increasingly seeking direct information for prevention, treatment, and emotional support regarding skin cancer on the Internet. Social media platforms appear to be used in different proportions for skin cancer awareness. Skin cancer prevention interventions delivered through social media may enhance their reach and relevance, especially among youth. In recent times, social media and the beauty industry go hand in hand [10]. Beauty brands, including dermatologic, represent many of the most-followed, active accounts on Instagram. Instagram is viewed by 1 billion users each month with 95 million posts and 3.5 billion “likes” per day. A total of 60% of users log in daily and 90% of users are less than 35 years of age. The U.S. has 110 million users alone; more users than any other country worldwide [29]. Dermatology content on Instagram is most prevalent among private offices, cosmetic brands, and to a lesser extent patient advocacy groups, with a notable lack of top dermatology journals and professional organizations maintaining accounts [11]. In a recent study by Jhawar and Lipoff which studied the potential of social media platforms in raising skin cancer awareness, they found that Instagram had the highest number of individual postings, and Twitter had the greatest social media reach [4]. Hashtags are keywords users can add to label their posts and that other users can then find by searching for those words. In 2018, Park et al. reported that among the top 20 hashtags related to medical and procedural dermatology on Instagram were “sun damage” with 73,332 results, “melanoma” with 68,743 and, “skin cancer” with 65,776 [34]. As of March 2021, those numbers are up to 261,000, 237,000, and 233,000, respectively. These numbers clearly suggest a growing interest in skin cancer and sun damage awareness. In fact, Basch et al. found out that a considerable portion of the Instagram posts focus on skin cancer prevention. The majority of the posts on this platform are images of people with considerable sun damage or post-operative images with a caption telling their story and advising people to wear sunscreen and avoid tanning. Their testimonials focus on delivering the facts in a compelling way that is simple to understand and raises awareness in a visual way. This indicates that Instagram could be used to promote health, particularly among young adults who represent a vast number of Instagram users and who are vulnerable to messages supporting risky behaviors that can elevate skin cancer risk such as tanning seen on social media [29].

#### 4.3.2. Twitter

Twitter is the most used platform within the scientific community and, as a result, has an emerging role in the dissemination of health information. This platform has shown broad applicability to public health surveillance and research. As an example, Twitter reached a maximum volume of conversations related to COVID-19 in one day with 548,152,410 tweets according to the WHO [12]. There is growing evidence that a highly mentioned paper in Twitter may reflect the quality of the paper and may be subject to a debate in journal clubs. Twitter data provides unique insight into tanning opinions, behaviors, and injuries not captured through traditional public health surveillance. Silva et al. reported that people use Twitter to communicate about their sun-related and skin cancer experiences and to share advice and information on this matter [13]. Overall, people use Twitter more frequently to discuss sunscreen and skin cancer, but not so often to communicate about other sun-protective behaviors or skin cancer prevention campaigns. Collection of tweets related to indoor tanning over a two-week period in 2013 revealed mention of “burn” in 4.75% of cases. Adverse health impacts in total (also including “cancer”, “danger”, “itch”, “pain”, and “aging”) were mentioned in less than 15% of cases, in spite of over 100 million followers potentially exposed to this messaging [11]. Comparably, Seidenberg et al. found that a total of 23,558 tweets that were tweeted in 2013 included keywords for indoor tanning and burning. Of these, 71.4% unambiguously described burns caused by tanning bed usage. Burn patterns among tanning bed users were further stratified and found that the most common sites of injury were the buttocks, followed by the face and chest [14]. Another study found that in over a two-week period, >150,000 posts on Twitter, reaching >100 million users, mentioned indoor tanning, with <5% of the tweets mentioning risks [15]. Due to Twitter’s extensive reach and relevance, dermatologists should consider extending its use for the communication of skin cancer-related information. There is a lack of emphasis and information about the adverse effects of tanning. It is important to identify these patterns of self-injurious behaviors, to target public health campaigns accordingly, and promote healthy and appropriate behaviors such as daily sunscreen use and indoor tanning cessation.

#### 4.3.3. Facebook

Facebook is the most used social media platform in the world with over 2.740 billion monthly active users worldwide and the highest monthly minutes spent per visitor [1]. Facebook is a popular site for knowledge dissemination related to dermatology, with dozens of professional societies and patient-centered groups maintaining large followings [11]. The level of public engagement with respect to dermatology content was recently found to be highest for educational posts, followed by interactive posts, news articles, and academic articles. Facebook pages carry a significant potential for skin cancer awareness. At present, organizations such as the Skin Cancer Foundation and the Melanoma Research Alliance have over 65,082 and 59,991 followers, respectively. This may be an indication that content with an informative purpose is of greater interest to Facebook users. Petukhova et al. reported that 63% of Facebook posts related to skin cancer focus on sharing personal experiences and providing words of encouragement, similar to a social support group [16]. Analysis of skin cancer prevention messages posted by 24 popular skin cancer groups on Facebook over a single year found that the majority of posts were risk-focused, with many using fear as the major motivational strategy [17]. This is consistent with literature supporting negative outcomes messages as more impactful than positive outcomes messages across the array of skin cancer prevention and risk behaviors. Noar et al. described the case of a young woman who posted a photo of herself on Facebook after undergoing treatment for skin cancer on her face, in which she cautioned viewers to avoid tanning beds and prolonged sun exposure. This post went viral and was reported by news media after it was shared on Facebook 50,000 times. Soon thereafter, online searches for skin cancer information reached near-record levels, demonstrating the capacity of social media messages to increase public engagement with prevention content as well as an opportunity for patients to spread skin cancer awareness with their own reflections, experiences, and regrets of past behaviors [9].

#### 4.3.4. YouTube

YouTube is the second most popular social media platform, with over 2.291 billion users worldwide [1]. A user’s ability to enter any search term and view related videos makes it a potentially useful tool to educate the public on health conditions. Digital videos appear to increase awareness and understanding of the risk factors and screening procedures related to skin cancer. They are a feasible way to increase general knowledge of cancer and sun protection. St Claire et al. found that the primary type of content related to sun protection on YouTube was 30% educational, 30% personal, and 30% user product review. The primary type of content related to skin cancer awareness was tied between 35% educational and 35% news/medical feature. Educational videos related to skin cancer were the predominant type of content (60%), with 25% of those educational videos deemed misleading [18]. Lauckner and Whitten compared Twitter, YouTube, and Facebook as sources of information on cancer risk reduction messages. Their research found that watching videos on YouTube lead to higher comprehension when compared with Twitter. Additionally, videos on YouTube led to stronger attitudes towards taking steps to reduce their cancer risk when compared with Facebook [19]. Similarly, Damude et al. found that YouTube videos on self-inspection of skin for melanoma patients were preferred as a supplement to receiving information from a physician and/or from written information. Furthermore, 79% of participants reported improved self-efficacy in performing self-skin inspections after watching the educational YouTube video [20]. Omission of adverse effects of tanning is also prevalent on YouTube. For instance, in a study by Hossler and Conroy, out of 72 videos pertaining to tanning beds, only 9 mentioned the possibility of burning, and 68% of the videos took an overall positive position on tanning [21]. Nonetheless, YouTube has a significant portion of popular videos related to dermatology that involve trained health care professionals. 

#### 4.3.5. TikTok

Although TikTok is relatively new, it has gained incredible popularity and has now over 689 million monthly active users [1]. With more than 2 billion total downloads since its debut, TikTok is the world’s fastest growing social media platform [22]. TikTok began as an app to create short comedy videos and dance clips, but as beauty tutorials and product demonstrations soon proved popular, so too did a professional opinion. Although TikTok’s potential has been demonstrated, its effects on skin cancer prevention are yet to be explored. Dermatologists have taken interest in TikTok’s ever-growing popularity. The duet feature on TikTok enables dermatologists to respond to another user’s clip, and interact with the original video, adding a new spin on the original creator’s upload. Duets have become a key element to interact and engage with the online community towards stopping skin care myths and the spread of misinformation. Challenges include viral video trends that encourage production of videos adhering to a general framework. Often these comprise choreography, comedy sketches, etc.; however, certain challenges encourage risky behaviors. For example, the “#tanningchallenge” encourages development of a natural tan; the “#tanningbedchallenge” encourages posting of videos from within a tanning bed and “#sunburnchallenge” collates videos of dramatically sunburned participants [23]. These trends can promote risky behaviors such as excessive UV exposure, pursuit of artificial or unrealistic aesthetic ideals, and tanning-bed use. According to Kassamali et al., the number of views for “skin cancer” on TikTok in September 2020 was 35 million, increasing to 43.2 in February 2021 [24]. Such trends allow dermatologists to understand patients’ health habits and concerns [35]. Increasing the number of board-certified dermatologists active on TikTok may result in the dissemination of more reliable medical information and healthy behaviors. Because of TikTok’s broad reach among adolescents and young adults, demographics in which tanning is highly prevalent, dermatologists should consider leveraging TikTok to communicate important messages aimed at sun protection and skin cancer awareness. Increasing the number of board-certified dermatologists active on TikTok may result in the dissemination of more reliable medical information and healthy behaviors. 

### 4.4. Influencers and Their Impact on Skin Cancer Prevention

Influencers are individuals who have a dedicated social media following and are used for social media marketing that uses endorsements and product mentions from these persons. Influencer marketing works because of the high amount of trust that social influencers have built up with their following, and recommendations from them serve as a form of proof of the reliability and quality of what they are advertising [36]. Currently, “skinfluencers” like Hyram Yarbro represent one of the most popular influencers with up to 6 million followers on TikTok. Dermatologists are among the physicians with the biggest social media presence, some of which are recognized as social media influencers [37]. Dr. Sandra Lee, also known as Dr. Pimple Popper, is one of the most popular dermatologists on social media, with up to 4 million followers on Instagram and 7 million subscribers on YouTube. Therefore, dermatologists also have the ability to affect patients’ health-related behaviors. As previously mentioned, celebrities also play a huge role in the influence of healthy behaviors. A few years ago, the Australian actor Hugh Jackman was diagnosed with a basal cell carcinoma (BCC) on his nose. This was his sixth BCC which required surgical intervention. With each new diagnosis, Jackman used his social media to warn the public of the dangers of sun exposure and advised them to use sun protection. By openly sharing his experience of diagnosis, excision, and recurrence of BCCs, Jackman has increased public awareness of skin cancer. This has been demonstrated by peaks in popularity of internet searches for the term basal cell carcinoma at the time of his social media posts [38]. Similarly, in January of 2021, Cardi B revealed that she was experiencing breakouts and dealing with dry skin, so to get help with the issue, she turned to her Twitter followers for advice who delivered within minutes [39]. These are some examples of how easy it is to find skin care tips on social media these days. It may be time to partner with influential people in order to take skin cancer awareness to the next level. It is the time for health to go viral.

## 5. Final Remarks

It is important to recognize that social media has become an increasingly important and readily available source of information to the public. The dermatology community may be able to use these venues to promote safer skin practices and broadcast appropriate health messages directly to those at highest risk. In today’s technological society, it is critical to understand and study the best form of communication on the different platforms. Specific platforms like Instagram, Twitter, Facebook, YouTube, and TikTok vary in originators of material, target demographics, messaging strategies, and reliability of information with regards to skin cancer, sun, and indoor tanning damage. Currently, trends with social media usage within dermatology indicate that Twitter and Facebook are the most used while Twitter tends to cover the most topics. The retweeting feature of Twitter is ideal for audience engagement as it allows propagation of information to new users and enables information cascades. Moreover, if a tweet goes viral, the speed and scale of both information spreading and audience engagement can be accelerated. Dermatologists should keep pace with the latest dermatological content on social media and examine its evolution as proper messaging has the ability to connect with millions worldwide. Physicians, scientists, and health care workers should form alliances with technology companies to develop creative ways to use social media to influence people with the right messages. It is crucial to develop messages that shift norms about ideals of appearance around a more healthy appearance rather than merely a beautiful appearance [40]. It is also important to consider the content of these messages; image-based messages appear to be more effective for skin cancer prevention [41]. The most engaging messages are didactic, appearance-based, myth-busting, use celebrity endorsements, and target self-exams. Given the increased incidence of tanning during the summer months, campaigns may be best implemented during this time. There should also be an increased use of social media to boost support, engagement, and advocacy for policies against indoor tanning. Efforts should be made to increase public awareness of the health implications caused by UV exposure and the importance of sunscreen use and sun protection.

## 6. Conclusions

Social media interventions have shown promise in skin cancer prevention and continue to escalate by the day, although retention is often low. To counter this limitation, dermatologists using social media as a means of skin cancer prevention should apply lessons from other domains. This study provides important insights into how social media may be used effectively for skin cancer awareness and prevention. A limitation of social media is the ability to quickly disseminate false information which can confuse people or lead them to the pursuit of risky behaviors. Responsible use of these tools can help to disseminate accurate information, generate relevant new scientific findings, share diagnostic, treatment, and follow-up protocols, as well as compare different approaches globally. Society relies on scientists and physicians to deliver fact-based information to the public. For this reason, it is our duty as physicians to be leaders in the conversations of social media and to guide correct and helpful information to the people looking for answers. Lastly, it is important to understand the type of support and education patients may need beyond the clinical setting, as well as to empower them to take a proactive approach to sun protection and the early detection and treatment of skin cancer. Further research is needed to evaluate the effectiveness and true impact of social media on meaningful and lasting behavior change for skin cancer prevention. 

## Figures and Tables

**Table 1:** Article characteristics in terms of type of article, targeted issue, and social media discussed.

Authors	Article Type	Targeted Skin Related Topic	Targeted Social Media Platform
Basch and Hillyer [5]	Short communication	Skin cancer	Instagram
Abbott et al. [6]	Letter to the editor	Tanning attitudes	Instagram, Twitter, Facebook
Falzone et al. [7]	Review	Skin cancer and indoor tanning	Twitter, Instagram, Facebook
Noar et al. [8]	Short communication	Skin cancer	Facebook
DeBord et al. [9]	Review	Skin cancer and indoor tanning, and others	Instagram, Twitter, Facebook, YouTube
Jhawar and Lipoff [10]	Commentary	Skin cancer	Instagram, Twitter, Facebook
Park et al. [11]	Editorial	Skin cancer, melanoma, sun damage, and others	Instagram
Vasconcelos Silva et al. [12]	Original article	Skin cancer and sun-related behaviors	Twitter
Seidenberg et al. [13]	Original article	Tanning bed burns	Twitter
Wehner et al. [14]	Letter to the editor	Indoor tanning and tanning health risks	Twitter
Petukhova et al. [15]	Original article	Skin cancer	Facebook
Nosrati et al. [16]	Research letter	Skin cancer and sun protection	Facebook
St. Claire et al. [17]	Commentary	Sun protection, skin cancer, and tanning	YouTube
Lauckner and Whitten [18]	Randomized control trial	Skin cancer	YouTube, Facebook, Twitter
Damude et al. [19]	Original article	Melanoma	YouTube
Hossler and Conroy [20]	Research letter	Indoor tanning	YouTube
Villa-Ruiz et al. [21]	Letter to the editor	Skin cancer and others	TikTok
Roche et al. [22]	Letter to the editor	Tanning, sunburn, and others	TikTok
Kassamali et al. [23]	Letter to the editor	Skin cancer and others	TikTok
Rahmani et al. [24]	Research letter	Skin cancer	Instagram

**Table 2:** Important statistics.

	Number (%)
**Article Types**	*n* = 20
Letter to the editor	5 (25%)
Original article	4 (20%)
Research letter	3 (15%)
Commentary	2 (10%)
Short communication	2 (10%)
Review	2 (10%)
Editorial	1 (5%)
Randomized control trial	1 (5%)
**Topics covered**	*n* = 20
Skin cancer	14 (70%)
Indoor tanning	4 (20%)
Tanning	2 (10%)
Melanoma	2 (10%)
Sun protection	2 (10%)
Sun-related behaviors	1 (5%)
Tanning bed burns	1 (5%)
Tanning health risks	1 (5%)
Sun damage	1 (5%)
Sunburn	1 (5%)
**Topics covered by each social media platform**	*n* = 10
Twitter	6 (60%)
Facebook	5 (50%)
Instagram	5 (50%)
YouTube	5 (50%)
TikTok	3 (50%)
**Most used social media platforms**	*n* = 20
Twitter	8 (40%)
Facebook	8 (40%)
Instagram	7 (35%)
YouTube	5 (25%)
TikTok	3 (15%)

## Data Availability

The data presented in this study are available in PubMed.
